# RNA binding motif protein 43 (RBM43) suppresses hepatocellular carcinoma metastasis by regulating Slug mRNA stability

**DOI:** 10.1016/j.gendis.2023.101192

**Published:** 2023-12-14

**Authors:** Yao Liu, Huan Feng, Qi Zhao, Xiao Liang, Ying Wang, Shuai Xiao, Suqin Shen, Jiaxue Wu

**Affiliations:** a State Key Laboratory of Genetic Engineering and School of Life Sciences, Fudan University, Shanghai 200438, China; b The First Affiliated Hospital, Cancer Research Institute, Department of Gastrointestinal Surgery, Hengyang Medical School, University of South China, Hengyang, Hunan 421001, China

RNA binding protein is a highly conserved protein family with the RNA binding domain to bind RNA and alter RNA metabolism and function.^1^ Abnormal expression of RNA binding protein is common in malignant tumors and connected to migration, invasion, death, and proliferation.^2^ RBM43, a member of the RNA binding protein family, was verified to be considerably down-regulated in hepatocellular carcinoma (HCC), indicating poor prognosis in HCC patients. RBM43 acted as a tumor suppressor that modulated CCNB1 expression to regulate the cell cycle, providing sufficient evidence for the significance of RBM43 in HCC.^3^ However, it is unknown if RBM43 has an impact on the process of HCC metastasis. Here, we discovered that Rbm43-deficient mice treated with diethylnitrosamine and carbon tetrachloride developed more intrahepatic and lung metastasis tumors. Consistently, the ability of migration and invasion was dramatically promoted in RBM43-deficient HCC cells. In terms of mechanisms, RBM43 suppressed HCC cell migration through the regulation of Slug mRNA stability. Additionally, in contrast to primary HCC and non-tumorous tissues, RBM43 expression was markedly decreased in metastatic HCC. In summary, our findings implied that RBM43 played a pivotal role in regulating the metastatic process of HCC, and highlighted the RBM43-Slug axis as a prospective HCC therapeutic target.

When we dissected mice after natural death with liver cancer induced by diethylnitrosamine and carbon tetrachloride, we found that compared with wild-type mice, more *Rbm43*-deficient mice developed ascites and metastatic lesions. As shown in Figure 1A, none of wild-type mice appeared ascites, while 4 out of 14 Rbm43-deficient mice produced ascites. 2 out of 12 wild-type mice had metastatic lesions, while 8 out of 14 Rbm43-deficient mice had metastatic lesions (Fig. 1B). Additionally, *Rbm43*-deficient mice with liver cancer also developed more metastatic nodules, which were dispersed around the surface of the large liver, lung, and kidney in comparison to wild-type mice (Fig. S1A). Hematoxylin and eosin staining was performed to verify the metastatic lesions in the lung and kidney (Fig. S1B). Together, our findings suggested that RBM43 might function as a suppressor of HCC metastasis *in vivo*.

**Figure 1:**
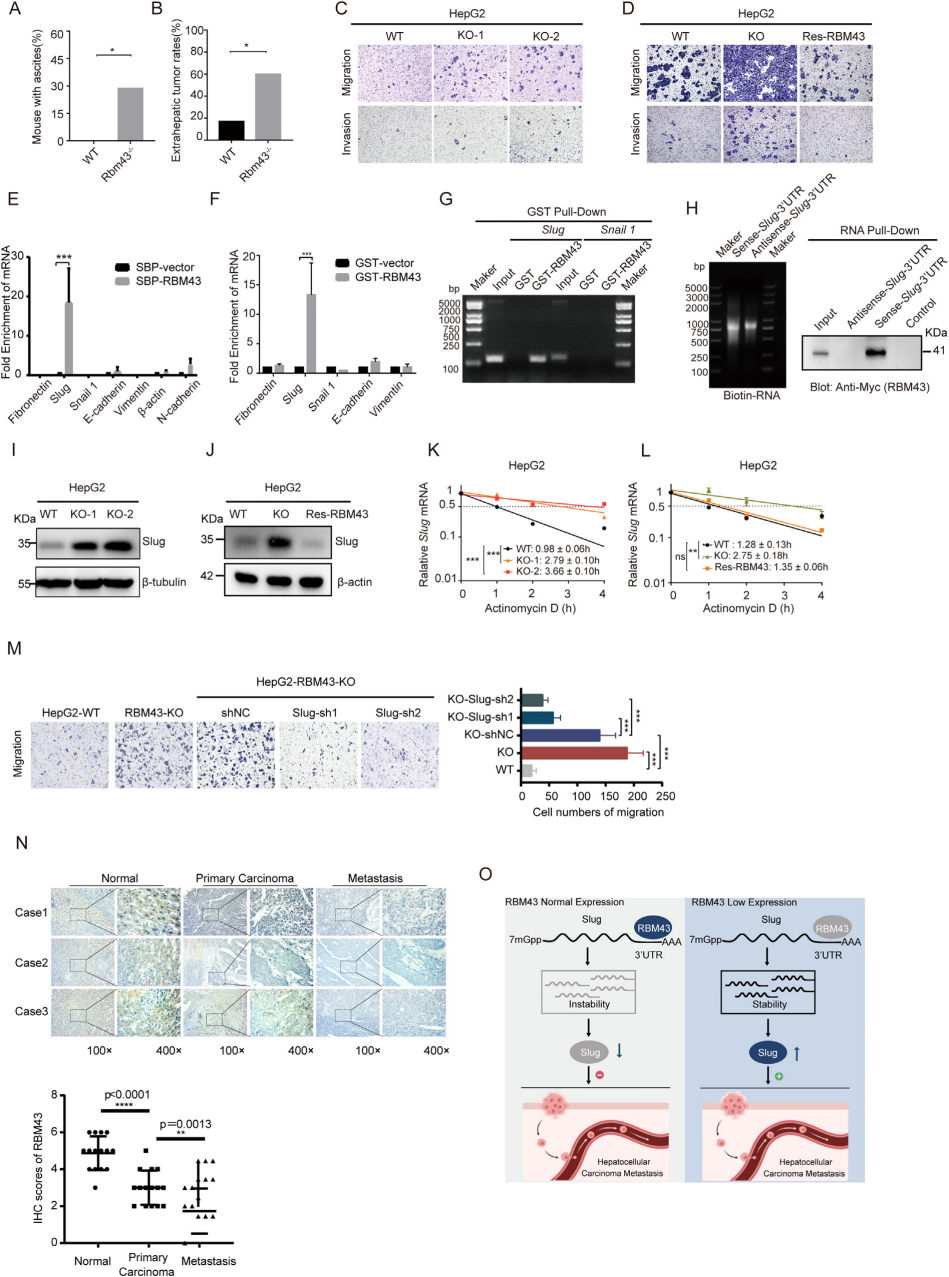
RBM43 suppresses hepatocellular carcinoma metastasis by regulating Slug mRNA stability. **(A)** After DEN-CCl_4_ treatment, the percentage of wild-type (*n* = 12) or *Rbm43*^*−/−*^mice (*n* = 14) with ascites was calculated. ^∗^*P* < 0.05. **(B)** After DEN-CCl_4_ treatment, the extrahepatic tumor rates of wild-type (*n* = 12) or *Rbm43*^*−/−*^ mice (*n* = 14) were calculated. ^∗^*P* < 0.05. **(C)** The effects of RBM43 deficiency on migration and invasion were measured by transwell assay in HepG2 cells. Representative images of migrated and invaded cells are shown. **(D)** The effects of RBM43-reconstitutions in RBM43 knockdown HepG2 cells on migration and invasion were measured by transwell assay. Representative images of migrated and invaded cells are shown. **(E)** RBM43 was specifically associated with *Slug* mRNA as analyzed by RNA immunoprecipitation assay. Flag-RBM43-associated RNAs were immunoprecipitated from Flag-RBM43 overexpressing Hep3B cells using streptavidin beads, and the RNA immunoprecipitation complex was analyzed by RT-qPCR using indicated primers as listed in Table S1, where β-actin was used as a negative control. ^∗∗∗^*P* < 0.001. **(F)** RBM43 was specifically associated with *Slug* mRNA as analyzed by GST pull-down assay. The complex pulled down by GST or GST-RBM43 was analyzed by RT-qPCR using indicated primers as listed in Table S1. ^∗∗∗^*P* < 0.001. **(G)** RBM43 was specifically associated with *Slug* mRNA as analyzed by DNA gel electrophoresis assay. Real-time PCR products after GST pull-down assay were analyzed through DNA gel electrophoresis to compare the intensity of *Slug* and *Snail1* enriched by GST or GST-RBM43. **(H)** RBM43 associated with 3′UTR of *Slug* mRNA as analyzed by RNA pull-down assay. The sense or antisense biotinylated-*Slug*-3′UTR transcript *in vitro* was generated as shown in the left panel. RBM43 was analyzed by Western blot using an anti-Myc antibody as shown in the right panel. **(I)** The protein level of Slug was examined in RBM43-deficient HepG2 cells by Western blot. **(J)** The protein level of Slug was examined by Western blot in RBM43-deficient HepG2 cells with RBM43-reconstitution. **(K)** The half-life of *Slug* mRNA was examined in RBM43-deficient HepG2 cells by actinomycin D treatment. ^∗∗∗^*P* < 0.001. **(L)** The half-life of *Slug* mRNA was examined in RBM43-deficient HepG2 cells with RBM43-reconstitution by actinomycin D treatment. ^∗∗^*P* < 0.01. **(M)** Migration capacities were measured by transwell assays in RBM43-deficient HepG2 cells with or without silencing Slug. Representative images of migrated cells are shown in the left panel. The histograms in the right panel show the mean numbers of migrated cells from three independent tests (mean ± standard deviation). ^∗∗∗^*P* < 0.001. **(N)** RBM43 protein expression was analyzed by immunohistochemistry staining using anti-RBM43 antibody in 15 pairs of metastatic HCC, primary HCC, and corresponding non-cancerous tissues. The upper panel shows the representative 3 cases of immunohistochemistry staining (magnification, × 100, × 400), and the lower upper shows the statistical analysis of immunohistochemistry staining results (paired two-tailed *t*-test was used). ^∗∗^*P* < 0.01, ^∗∗∗∗^*P* < 0.0001. **(O)** The hypothetical working model. The diagram indicated that RBM43 was associated with *Slug* mRNA 3′UTR and negatively regulated its stability and then suppressed hepatocellular carcinoma metastasis. DEN, diethylnitrosamine; CCl_4_, carbon tetrachloride.

To investigate the effect of RBM43 on HCC metastasis further, we constructed RBM43-deficient HepG2 and QGY-7703 cells through CRISPR-Cas9 technology and successfully reconstituted RBM43 in knockdown HCC cell lines by lentivirus infection (Fig. S2A–D). We demonstrated that RBM43 deficiency markedly improved the ability of HepG2 cells in migration and invasion, and RBM43-reconstitution significantly inhibited the ability in migration and invasion compared with RBM43-deficient HepG2 cells (Fig. 1C, D; Fig. S3E, F). We also validated this result in QGY-7703 cells (Fig. S3A–D).

Previously, we profiled the transcriptomes of SMMC-7721 cells with RBM43 deficiency using RNA-Seq and found more than 400 dysregulated genes.^3^ Interestingly, we found that Slug, which played a critical role in cell migration and invasion,^4^ was up-regulated after RBM43 knockdown (data not shown). As an RNA binding protein, RBM43 can modulate RNA stability, raising the potential that RBM43 may participate in cell migration and invasion via modulating the mRNA stability of *Slug.* As the RNA immunoprecipitation experiments demonstrated, *Slug* mRNA was dramatically enriched in the SFB-RBM43 complexes (Fig. 1E), implying that RBM43 might interact with *Slug* mRNA. Consistent with the immunoprecipitation results, *Slug* mRNA, not other EMT markers, was dramatically enriched in the GST-RBM43 complexes (Fig. 1F, G). In the RNA pull-down assay, Myc-RBM43 was successfully pulled down by sense *Slug*-3′UTR, not antisense *Slug*-3′UTR and control. Collectively, these findings disclosed that RBM43 was associated with *Slug* mRNA via its 3′UTR (Fig. 1H).

Next, we compared the expression of Slug in wild-type or RBM43-deficient cells. *Slug* mRNA and protein levels were significantly raised in HepG2 and QGY-7703 cells with RBM43-deficiency in comparison to wild type (Fig. S4A, B; Fig. 1I). Similarly, we discovered that *Slug* mRNA was notably increased in tumors from mice with *Rbm43*-deficiency in comparison to wild-type mice (Fig. S4C). Then we performed an actinomycin D assay to compare *Slug* mRNA stability. *Slug* mRNA had a longer half-life in HepG2 and QGY-7703 cells with RBM43-deficiency than in wild-type cells (Fig. 1K; Fig. S4D). Moreover, RBM43-reconstitution of RBM43-deficient HepG2 cells remarkably decreased the *Slug* mRNA and protein expression (Fig. 1J) and shortened the half-life of *Slug* mRNA (Fig. 1L). These findings suggested that RBM43 acted as a negative regulator of *Slug* mRNA stability.

*Slug* is a well-known tumor metastasis-associated positively regulated gene.^4^ RBM43 regulates the stability of *Slug* mRNA, providing a possibility that RBM43 suppresses cell migration depending on Slug. We interfered Slug protein in RBM43-deficient HepG2 by shRNA (Fig. S4F) and examined cell migration to verify this hypothesis. Knockdown of Slug dramatically inhibited the migration of RBM43-deficient HepG2 cells (Fig. 1M). These results suggested that RBM43 suppressed the HCC cell migration through the regulation of Slug.

Since RBM43 inhibited HCC cell migration, it advised us to investigate the RBM43 expression in primary lesions and metastatic lesions of HCC varied from those in corresponding normal tissues. We assessed the level of RBM43 expression in a tissue array made up of 15 pairs of primary HCC, metastatic HCC, and corresponding normal tissues through immunostaining with anti-RBM43 antibody. In comparison with corresponding non-cancerous tissues, RBM43 is dramatically down-regulated in primary HCC, which is consistent with our previous results. Additionally, when compared with primary HCC, the expression of RBM43 in metastatic HCC is significantly down-regulated (Fig. 1N). These findings provided more evidence that RBM43 was critical for HCC metastasis.

As studied in this article, we found a novel role for RBM43 in HCC metastasis. We showed that, in contrast to primary HCC and corresponding non-tumorous tissues, metastatic HCC had an obvious decrease in RBM43 expression, and low expression of RBM43 increased the stability of *Slug* mRNA, then eventually promoted HCC metastasis (Fig. 1O). Slug expression in cancer was strictly controlled at the transcriptional, post-transcriptional, translational, and post-translational stages. Among these, post-transcriptional control stands out as a key way to modulate Slug expression. Our findings revealed a novel mechanism of *Slug* regulation mediated by RBM43 at the post-transcriptional level and suggested that the regulation of *Slug* mRNA stability is more complex. It remains unclear if mRNA modifications, such as N6-methyladenosine and polyadenylation, are possible post-transcriptional regulatory mechanisms for the expression and function of *Slug* in addition to mRNA stability.^5^

## Author contributions

YL and HF performed most of the experiments. YL, HF, and SS collected and analyzed the data. QZ, XL, and YW provided advice about the experiments. SX collected clinical hepatocellular carcinoma samples and conducted immunohistochemically analysis of RBM43. JW and SS conceived the study, provided overall guidance, and contributed to the manuscript's completion. All authors contributed to and approved the manuscript.

## Funding

This work was supported by grants from the 10.13039/501100001809National Natural Science Foundation of China (No. 82173269 to SS, 81,972,712 to JW).

## Conflict of interests

The authors have declared that no conflict of interest exists in this work.
