# Supplementary material for: RNA binding motif protein 43 (RBM43) suppresses hepatocellular carcinoma metastasis by regulating Slug mRNA stability
Source: Genes Dis. 2023 Dec 14;11(6):101192. doi: 10.1016/j.gendis.2023.101192 (PMC11298866; doi:10.1016/j.gendis.2023.101192)
Supplement: Multimedia component 1 [file mmc1.pdf]

Supplementary data for

RNA binding motif protein 43 (RBM43) Suppresses hepatocellular carcinoma metastasis by regulating Slug mRNA stability

Yao Liu[a,1], Huan Feng[a,1], Qi Zhao[a], Xiao Liang[a], Ying Wang[a], Shuai Xiao[b,**], Suqin Shen[a,*], Jiaxue Wu[a,*]

*Corresponding author. Email: Jiaxue Wu, jiaxue@fudan.edu.cn; Suqin Shen, shensq@fudan.edu.cn; Shuai Xiao, xiaoshuai1982@hotmail.com.

This file includes:

Supplementary Fig. S1 to S4
Supplementary Table. S1
Methods and Materials
Abbreviations

Supplementary Fig. S1 to S4

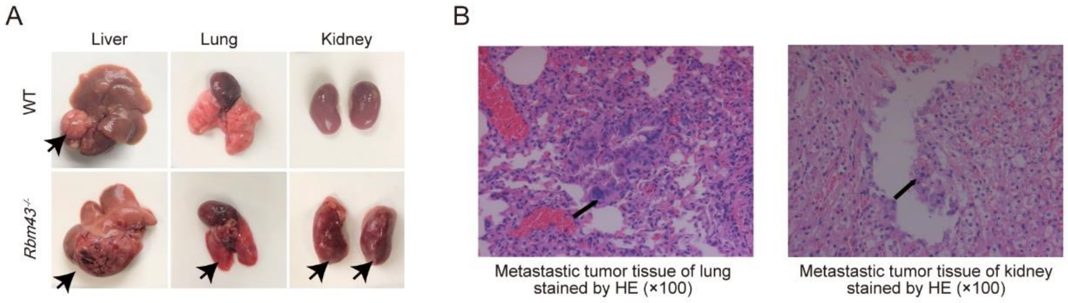

Figure S1 Loss of *Rbm43* promoted tumor metastasis in drug-induced hepatocellular carcinoma mice. **(A)** Representative images of liver, lung and kidney from wild-type (n=12) or *Rbm43*$^{-/-}$mice (n=14). Tumor nodules on different organ are indicated by arrows. **(B)** HE staining of tumor tissue for pathologic analysis. Histopathological changes were observed under a light microscope. Tumor infiltration of different organ indicated by arrows. Images captured at 100× magnification.

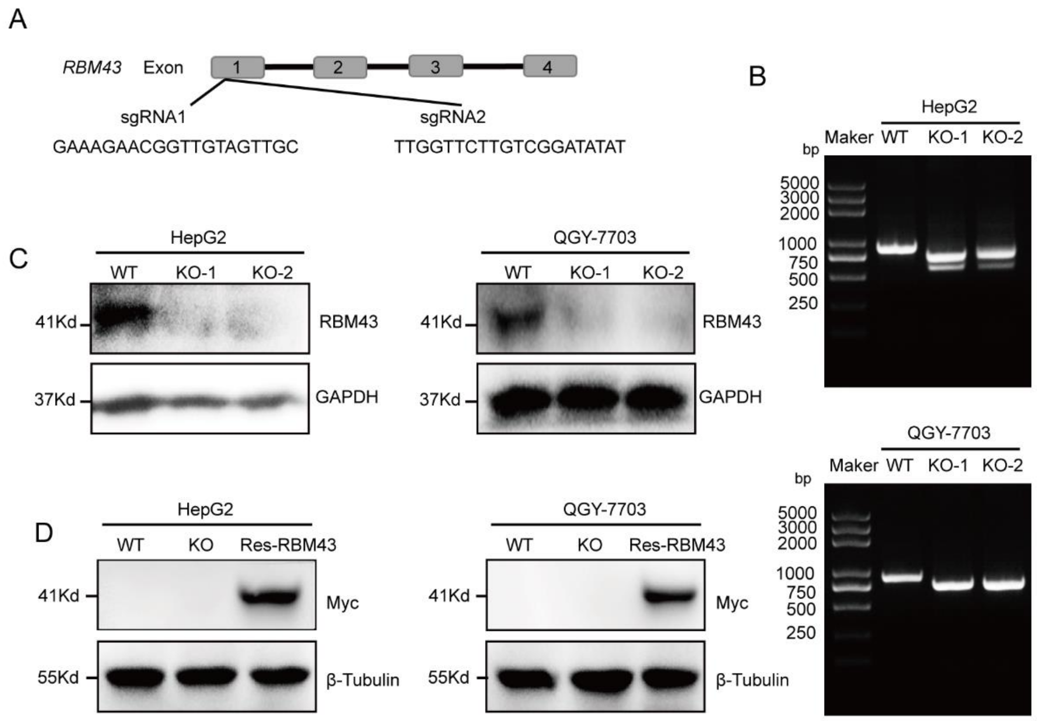

Figure S2 Identification of RBM43-deficient and RBM43-reconstituted hepatocellular carcinoma cell lines. **(A)** Diagram of two sgRNAs targeting the first exon of *RBM43*. **(B)** Identification of RBM43-deficient clones in QGY-7703 and HepG2 HCC cells by DNA gel electrophoresis. **(C)** Identification of RBM43-deficient clones in QGY-7703 and HepG2 HCC cells by western blot with RBM43 antibody. **(D)** Reconstitution of Myc-RBM43 in RBM43-deficient HCC cells identified by western blot with anti-Myc antibody.

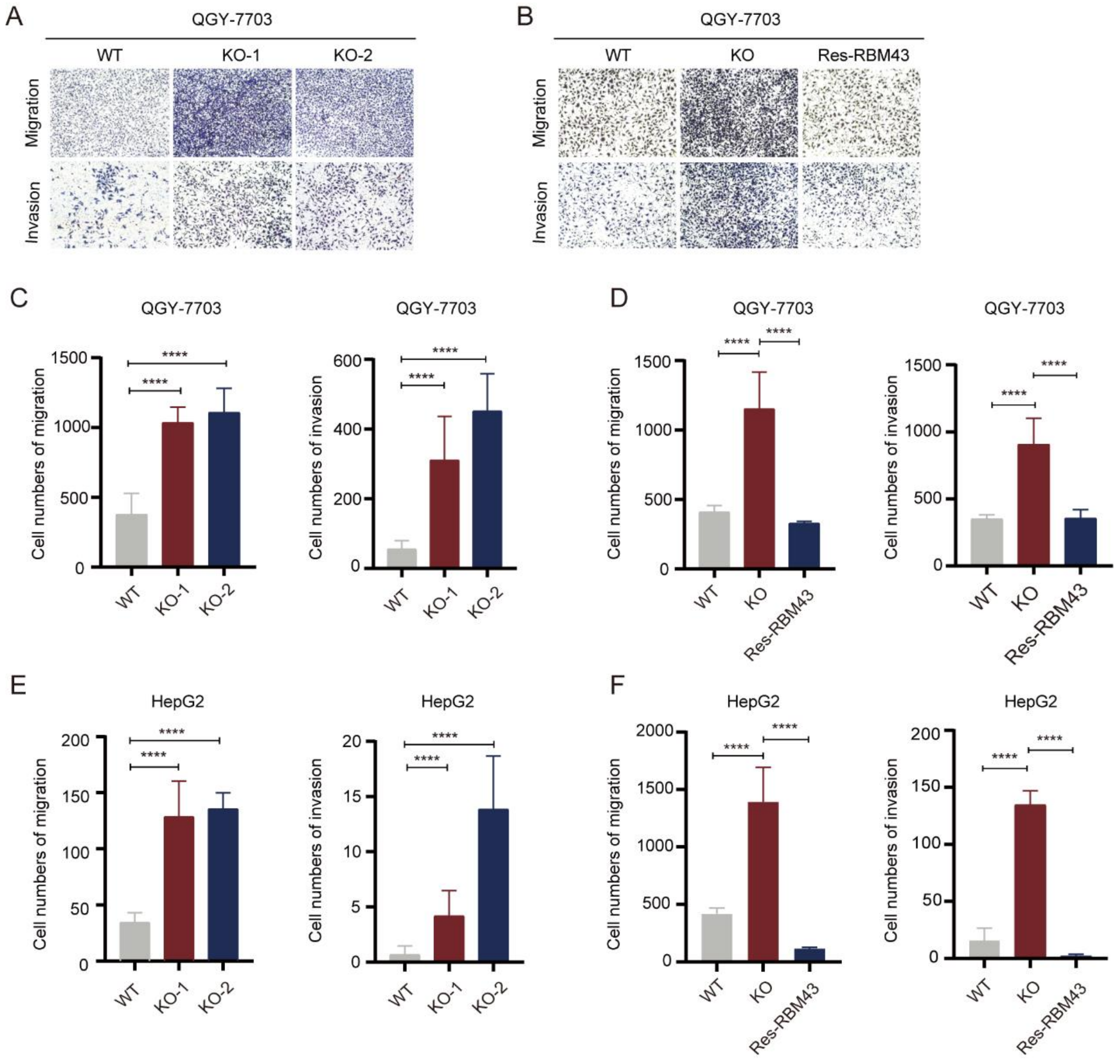

Figure S3 RBM43 regulated HCC cell migration and invasion. **(A)** The effects of RBM43-deficiency on migration and invasion were measured by transwell assay in QGY-7703 cells. Representative images of migrated and invaded cells are shown. **(B)** The effects of RBM43-reconstitutions in RBM43 knockdown QGY-7703 cells on migration and invasion were measured by transwell assay. Representative images of migrated and invaded cells are shown. **(C-F)** The histograms show the mean numbers of migrated and invaded cells from three independent tests (mean ± s.d.). ***P < 0.0001.

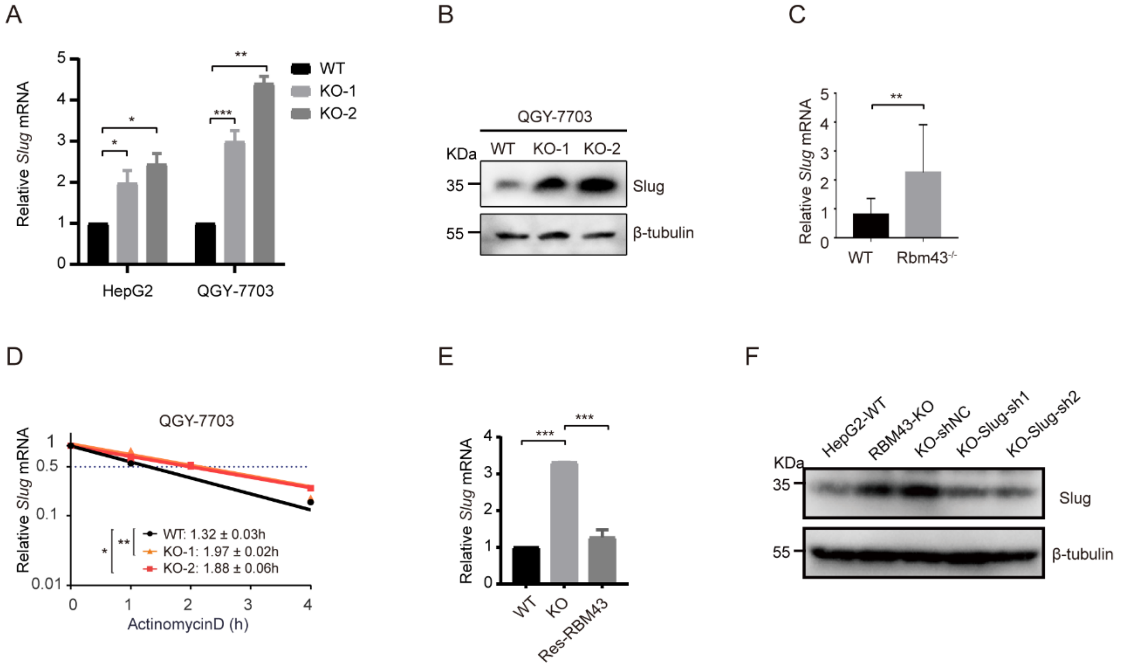

Figure S4 *Slug* mRNA stability was increased in RBM43 deficient cells. **(A)** The mRNA level of *Slug* was examined in RBM43-deficient HepG2 cells or QGY-7703 cells by RT-qPCR. *P < 0.05, **P < 0.01, ***P < 0.001. **(B)** The protein level of Slug was examined in RBM43-deficient QGY-7703 cells by western blot. **(C)** *Slug* mRNA enrichment was measured in DEN-CCl_4 treated wild-type mice (Male, n=12) or *Rbm43*$^{-/-}$ mice (Male, n=14) by real-time PCR and normalized to mouse GAPDH. **P < 0.01. **(D)** Half-life of *Slug* mRNA was examined in RBM43-deficient QGY-7703 cells

by Actinomycin D treatment. ***P < 0.001. **(E)** The mRNA level of *Slug* was examined in RBM43-deficient HepG2 cells with RBM43-reconstitution. ***P < 0.001. **(F)** Knockdown of Slug by shRNA in RBM43-deficient HepG2 cells. The expression of Slug was analyzed by western blot using anti-Slug antibody.

Table S1

The Real-Time PCR primers used in this study are listed below.

Gene Name	Sequence（5’-3’）
Slug-qPCR-F	ACAACTGGAGGAAGAGCAGTCAGTTAGAC
Slug-qPCR-R	GTGTTGTTAGTCACAAAGGCGAAGTC
mSlug-qPCR-F	TCTGTGGCAAGGCTTTCTCCAG
mSlug-qPCR-R	TGCAGATGTGCCCTCAGGTTTG
E-cadherin-qPCR-F	CACCCTGGCTTTGACGCCGA
E-cadherin -qPCR-R	AAAATTCACTCTGCCCAGGACGCG
β2-MG -qPCR-F	ATGAGTATGCCTGCCGTGTGAAC
β2-MG -qPCR-R	TGTGGAGCAACCTGCTCAGATAC
18S-qPCR-F	AAACGGCTACCACATCCAAG
18S -qPCR-R	CCTCCAATGGATCCTCGTTA
β-actin-qPCR-F	AAGGCCAACCGCGAGAAGAT
β-actin-qPCR-R	CACAGCCTGGAAGCAACGTAC
mGAPDH-qPCR-F	GTCATCCCAGAGCTGAACG
mGAPDH-qPCR-R	TCATACTTGGCAGGTTTCTCC
Vimentin-qPCR-F	GACGCCATCAACACCGAGTT
Vimentin-qPCR-R	CTTTGTCGTTGGTTAGCTGGT
N-cadherin-qPCR-F	CGCCATCCAGACCGACCCAA
N-cadherin-qPCR-R	GTCGATTGGTTTGACCACGGTGAC
Snail-qPCR-F	TCGGAAGCCTAACTACAGCGA
Snail-qPCR-R	AGATGAGCATTGGCAGCGAG

Materials and Methods

Human samples

A total of 15 pairs of primary and metastatic HCC and corresponding normal liver tissue samples after surgical resection were obtained from the First Affiliated Hospital of South China University (Hunan Province, China). All patients were diagnosed with hepatocellular carcinoma accompanied with metastatic lesion in surgery or confirmed by postoperative histopathological examination. The First Affiliated Hospital of South China University's Ethics Committee authorized the use of human tissues with informed consent.

Plasmids, antibodies, and other materials

All the plasmids including PGEX-4T-1-RBM43 (GST-tagged), PCMV-Myc-RBM43 (Myc-tagged), PLVX-puro-RBM43 (Myc-tagged), modified pRIES2-EGFP-RBM43 (SFB-tagged, SFB refers to S-protein/Flag/SBP) were successfully cloned as our previous work described and saved in our laboratory. Sense and antisense 3′UTRs of Slug (NCBI Accession NC_ 000008.11) were cloned into the PsiCHECK-2 vector for reverse transcription templates. The shRNA targeting Slug was cloned into the PCDH-U6-SHRNA vector for Slug silencing.

Antibodies including anti-β-actin (T40104), anti-GAPDH (M20006), anti-β-tubulin (M20005), anti-Flag (M20008) antibodies from Abmart (Shanghai, China), anti-Myc (GNI4110-MC) from GNI (Kyoto, Japan), anti-Slug (C19G7) produced from Cell Signaling Technology (Beverly, MA, USA), anti-RBM43 (ab108035) for western blot

purchased from abcam (Cambridge, MA, USA) and anti-RBM43 antibody (HPA038204) for IHC staining ordered from Sigma (St Louis, MO, USA) were all kept in a -20°C refrigerator. We selected streptavidin agarose beads from Smart-Lifesciences (Changzhou, China).

Cell Culture & Transfection

We ordered HEK-293T cells and Human-derived HCC cell lines (HepG2, QGY-7703, Hep3B) from Shanghai Cell Bank of Chinese Academy of Sciences (Shanghai, China). Cells were grown in DMEM comprising 10% FBS, 1% streptomycin and penicillin with 37°C.

For cell transfection, we transfected the plasmid to cells with PEI reagent (Polysciences, IL, USA) based on the manufacturer instructions. Remembered to change serum-free medium to fresh medium with 10% FBS six hours after transfection.

Migration and invasion assay

QGY-7703 or HepG2 cell lines cultured to logarithmic phase were digested and collected, removed the supernatant and washed cells once with 1×PBS and DMEM followed. 100μl serum-free DMEM with 2×10^4 QGY-7703 or 5×10^4 HepG2 cells were softly dropped into the chamber, then put the chamber into a transwell plate (Corning, New York, USA) containing fresh medium containing 10% FBS (pay attention to avoid large bubbles). After 36h culturing, the cells were fixed in 800μl 4% paraformaldehyde for 30min and stained with crystal violet at 0.2% concentration for 2h. Washed the chamber gently with clear water for several times and carefully wiped off unsuccessfully migrated cells with 75% ethanol dipped with cotton swab. After

photographing under a microscope, we counted and analyzed statistically the positive cells stained in purple in 8-10 random fields by ImageJ software. As for invasion experiments, chambers need to be pretreated with 1:20 Matrigel (BD Biosciences, CA, USA) dissolved in DMEM in 37°C incubator for 1h.

Generation of RBM43-deficient and reconstituted cells lines

CRISPR-CAS9 gene editing technology was adapted in this paper to construct RBM43 knockout HCC cell lines. Two sgRNAs (sgRNA1:GAAAGAACGGTTGTAGTTGC; sgRNA2:TTGGTTCTTGTCGGATATAT) designed to target the first exon of RBM43 gene were constructed onto epiCRISPR vectors according to the sgRNA design website: http://crispr.mit.edu. Then epiCRISPR vector carrying this two sgRNA was transfected into HepG2 or QGY-7703 cells. After 1 mg/ml puromycin screening and the method of limited dilution, we extracted the genomic DNA from every single clone following the manuals of the Genomic DNA Extraction Kit (Beijing, China), which was subsequently verified by PCR and DNA gel electrophoresis, eventually we obtained the correct clone (insertion or deletion of non-integer multiples of 3 bases) by sanger sequencing.

On the other hand, we used a lentiviral infection system to construct RBM43-reconstituted cells lines or Slug-silencing cell lines. Taking the construction of RBM43-reconstituted cells lines as an example, we co-transfected PLVX-myc-RBM43 with S6 and S7 into HEK-293T cells to package lentiviruses expressing RBM43 protein. At 48h post-transfection, we collected and filtered the cell medium with 0.45μM filters to obtain packaged lentivirus, which were used to infect HepG2 and QGY-7703 cells. We eventually obtained the RBM43-reconstituted cell lines through western blot by Myc-

labeled antibody.

Ribonucleoprotein immunoprecipitation

Ribonucleoprotein immunoprecipitation were carried out as we described before. [1]

In vitro RNA pull-down assay

In order to produce linearized templates for transcription in vitro, the following primers were synthesized (5'-3'): Sense-T7-Slug-3′UTR-F (TAATACGACTCACTATAGGGTGACGCAATCAATGTTTAC), Sense-Slug-3′UTR-R (TTTTTTTCTTGTTAACAAAC); Antisense-T7-Slug-3′UTR-F (TAATACGACTCACTATAGGTTTTTTTCTTGTTAACAAAC), Antisense-Slug-3′UTR-R (GTGACGCAATCAATGTTTAC). The following experimental steps of were operated as stated in the former article.[1]

RNA half-life Assay

Cells in good condition were seeded in cell plates with 12 wells at 70% confluence. After treatment with 10μg/ml of Actinomycin D (Sigma, St Louis, MO, USA) respectively for 0h, 0.5h, 1h, 2h, 3h, 4h, we collected cells, then washed them with 1× PBS and resuspended them with 1ml trizol for RNA extracting. We performed real-time PCR using ribosomal 18S RNA as internal standard.

RT-qPCR

Trizol (Thermo Fisher Scientific, CA, USA) were used to extract total RNA with the kit instructions, which were measured by nanodrop (Thermo Fisher Scientific, CA, USA) for its concentration and quality. The procedures for reverse transcription of RNA

and subsequent real-time quantitative fluorescence PCR were provided in the materials and methods section of the earlier paper. [1] Primers used in Real-Time PCR were given in Table S1.

DEN/CCl_4 mouse model

We constructed the DEN/CCL_4 mouse model according to the method stated in the former publication.[1]

Western blot

Proteins were separated through SDS-PAGE and transferred to PVDF membranes via a transfer system (Bio-Rad, CA, USA). Incubating the membranes with indicated primary antibodies at a recommended concentration overnight at 4°C after incubation with blocking buffer, followed by a 30-minute incubation with secondary antibodies that have been conjugated with HRP at room temperature. With the use of the ECL chemiluminescence imaging system, the results were made visible.

Histological analysis and immunostaining

Tissue samples from Human or mouse were collected with immediate fixing in 4% formaldehyde for 48 h. After embedding by paraffin and sectioning, liver sections were prepared for immunostaining with indicated antibody or staining with hematoxylin and eosin (H&E), all steps were carried out under the standard techniques we have described previously.[1] For immunohistochemical analysis, intensity of RBM43 staining was assessed on a scale of four-point (0_3 score).[2] Following criteria were used to rate the percent distribution of staining: less than 10% positive cells (0 score); 10%-40%

positive cells (1 score); 40%-70% positive cells (2 score); more than 70% positive cells (3 score). The results of the scoring based on the intensity and range of staining were summed to give a total staining score for RBM43.

Statistical Analysis

Regarding statistical analysis, GraphPad Prism Software (version 7.0) was applied in this study. The two-tailed Student's t-test was adopted when analyzing our data, and the thresholds for statistical significance were established at *P<0.05,**P<0.01, and ***P<0.001.

Abbreviations

RBM43 RNA binding protein 43;

DEN Diethylnitrosamine;

CCl_4 Carbon tetrachloride;

HCC Hepatocellular carcinoma;

EMT Epithelial-mesenchymal transition;

CCNB1 Cyclin B1;

RT-qPCR Real Time Flurocent Qualitative PCR;

IP Immunoprecipitation;

UTR Untranslated region;

Supplementary Reference

1. Feng H, Liu J, Qiu Y, et al. RNA-binding motif protein 43 (RBM43) suppresses hepatocellular carcinoma progression through modulation of cyclin B1 expression. Oncogene. 2020; 39(33): 5495-5506.

2. Yoo S S, Carter D, Turner B C, et al. Prognostic significance of cyclin D1 protein levels in early-stage larynx cancer treated with primary radiation. International Journal of Cancer. 2000; 90(1): 22-28.
